# MIDAS (Migraine Disability Assessment): a valuable tool for work-site identification of migraine in workers in Brazil

**DOI:** 10.1590/S1516-31802002000400006

**Published:** 2002-07-07

**Authors:** Yara Dadalti Fragoso

**Keywords:** MIDAS, Migraine, Headache, MIDAS, Cefaléia, Enxaqueca

## Abstract

**CONTEXT:**

MIDAS was developed as a fast and efficient method for identification of migraine in need of medical evaluation and treatment. It was necessary to translate MIDAS, originally written in English, so as to apply it in Brazil and make it usable by individuals from a variety of social-economic-cultural backgrounds.

**OBJECTIVE:**

To translate and to apply MIDAS in Brazil.

**SETTING:**

Assessment of a sample of workers regularly employed by an oil refinery.

**SETTING:**

Refinaria Presidente Bernardes, Cubatão, São Paulo, Brazil.

**PARTICIPANTS:**

404 workers of the company who correctly answered a questionnaire for the identification and evaluation of headache. When the individual considered it to be pertinent to his own needs, there was the option to answer MIDAS as well.

**METHODS:**

MIDAS, originally written in English, was translated into Brazilian Portuguese by a neurologist and by a translator specializing in medical texts. The final version of the translation was obtained when, for ten patients to whom it was applied, the text seemed clear and the results were consistent over three sessions.

**MAIN MEASUREMENTS:**

Prevalence and types of primary headaches, evaluation of MIDAS as a tool for identification of more severe cases.

**RESULTS:**

From the total of 419 questionnaires given to the employees, 404 were returned correctly completed. From these, 160 persons were identified as presenting headaches, 44 of whom considered it worthwhile answering MIDAS. Nine of these individuals who answered MIDAS were identified as severe cases of migraine due to disability caused by the condition. An interview on a later date confirmed these results. Three were cases of chronic daily headache (transformed migraine) and six were cases of migraine.

**CONCLUSIONS:**

MIDAS translated to Brazilian Portuguese was a useful tool for identifying severe cases of migraine and of transformed migraine in a working environment. The workers did not consider MIDAS to be difficult to answer. Their high level of voluntary participation demonstrates that this medical condition was of real interest among the workers, whether they were sufferers or not.

## RESUMO

**CONTEXTO:**

MIDAS foi desenvolvido como um método rápido e eficaz na identificação de casos de enxaqueca para orientar auxílio médico e tratamento. Tornou-se necessário traduzir o MIDAS, originalmente escrito em inglês, para aplicar no Brasil para que pudesse ser utilizado por indivíduos de vários níveis socioeconômicos e culturais.

**OBJETIVO:**

Traduzir e aplicar MIDAS no Brasil.

**TIPO DE ESTUDO:**

Avaliação de uma amostra de indivíduos regularmente empregados em uma refinaria de petróleo.

**LOCAL DE EXECUÇÃO:**

Refinaria Presidente Bernardes, Cubatão, SP.

**PARTICIPANTES:**

404 funcionários da empresa que responderam corretamente a um questionário para identificação e avaliação de cefaléia. Quando considerado pertinente, o funcionário tinha a opção de preencher também o MIDAS.

**MÉTODOS:**

MIDAS, originalmente escrito em inglês, foi traduzido para português do Brasil por uma neurologista e por um tradutor especializado em textos médicos. A versão final da tradução foi obtida quando, em 10 pacientes consecutivos a quem foi aplicada, o texto foi considerado claro e os resultados foram consistentes em três sessões.

**VARIÁVEIS ESTUDADAS:**

Prevalência e tipos de cefaléias primárias, avaliação do MIDAS como uma ferramenta de identificação de casos mais graves.

**RESULTADOS:**

Dos 419 questionários distribuídos, recolhidos e respondidos no mesmo dia, 404 retornaram completamente preenchidos. Destes, 160 pessoas foram identificadas como portadoras de cefaléia, 44 delas considerando adequado responder também ao MIDAS. Nove destes indivíduos que responderam ao MIDAS foram identificados como casos graves de enxaqueca devido à incapacidade produzida pela doença. Uma entrevista posterior e individual confirmou estes dados.

**CONCLUSÃO:**

MIDAS traduzido para o português do Brasil foi uma ferramenta útil na identificação de casos graves de enxaqueca em um ambiente de trabalho. Os funcionários não consideraram que MIDAS fosse difícil de responder. O elevado índice de participação voluntária dos funcionários demonstrou que esta condição médica era de interesse entre os trabalhadores, portadores ou não de cefaléia.

## INTRODUCTION

Migraine is essentially an episodic primary headache syndrome, moderate to severe in intensity, which is able to induce functional disability in a large number of cases. There is no epidemiological data from large population studies in Brazil, but it is generally accepted that the prevalence of migraine is of the order of 18% among females and 6% among males.^1^ Most sufferers have high frequency of attacks during the productive years of their lives, disrupting their capacity to work on the migraine attack days. Even if not absent from work, these sufferers have lower productivity during the headache attacks. A recent survey in Brazil showed that in a particular company, up to US$ 126.00 was potentially lost annually with each migraine worker.^2^ Recently, Brazilian surveys on the prevalence and impact of migraine have shown that this condition is not only highly prevalent, but indeed very disabling.^3-6^

Starting in 1998, a group of medical specialists created and implemented a questionnaire to assess the impact of disability in mi- graine.^7-9^ Usually, the reason why a patient seeks medical attention is the pain in the migraine attack. Likewise, the pain is the usual symptom treated by the doctor. However, it is the disability created by the migraine attack as a whole that will interfere the most in the productive life of an otherwise healthy individual and that will ultimately determine the proposals for continuous treatment.^10-12^

The questionnaire thus created, named MIDAS for "Migraine Disability Assessment", has proven to be a useful tool in identifying migraineurs with different degrees of headache-related disability. Furthermore, this questionnaire is brief, simple to use, consistent, highly reliable, and correlates with physicians' clinical judgment. MIDAS may provide a practical tool for helping to implement treatment recommendations for migraine, according to the Headache Consortium Guidelines of several countries.^13,14^

MIDAS has been shown to keep good correlation between the score and the medical needs of the patient. The reliability of this set of questions was tested in two countries, both English-speaking nations (United Kingdom and United States of America).^15^ There are now ten translations into other languages available online on the internet. Methods for translation and application of such international versions of MIDAS are not clearly specified.

Although Portuguese from Portugal is one of the languages into which MIDAS has been translated, Brazilian Portuguese is not on the list of present translations. The language similarities between the two countries may be sufficient to provide mutual understanding, but nonetheless the delicate matter of colloquial usage may influence the results. The questions must be clearly comprehended by the patients, who should be able to answer MIDAS without assistance from health workers.

The author has endeavored to translate and to apply MIDAS in Brazil, in such a way that, as originally intended by Lipton^7^ and Stewart et al.,^8^ the patients themselves may apply the questionnaire. By answering MIDAS without the influence of a doctor or other health professional, the possibility of outside interpretation and bias is avoided. Cultural background should not become a barrier to applying MIDAS. The individual is therefore empowered to access MIDAS through a web site and, using the scales of measurements and recommendations, seek out specialized medical help if such is the case.

## METHODS

MIDAS, originally written in English, was translated into Brazilian Portuguese by a Brazilian neurologist and by a native speaker of English working as a translator specializing in medical texts. The final version of the translation was obtained when, for ten consecutive patients to whom it was applied, the text seemed clear and the results were consistent over three sessions. The only difficulty in understanding any of the points of the questionnaire was related to the concept of "90 days", i.e. the patients seemed to naturally think on a 30-day basis. After discussing this point with the patients themselves, it seemed necessary to emphasize the need to consider 90 subsequent days and not 30 days multiplied by three. Therefore, in a further version of the translation, the concept of 90 days was underlined and insisted upon with more emphasis that in the original English version of MIDAS. No major alteration in the English version of MIDAS was required.

This final version was then taken to the Refinaria Presidente Bernardes, a state-owned oil refinery in Cubatão, in the State of São Paulo, for application to a large population of workers from a variety of cultural, educational and social backgrounds. The Department of Occupational Medicine of this company was most helpful in setting up the arrangements for our work. An initial questionnaire regarding the presence or absence of headache and its characteristics was handed out to each individual entering the company's restaurant on a particular day. Participation was voluntary, as was individual identification. By handing out the questionnaire to the employees in this way, the author was able to ensure that participants were filling out the form without medical intervention. All forms were collected after the meal. For those who presented headaches, MIDAS was offered as an extra tool for assessing the impact of the headache on that person.

## RESULTS

From the total of 419 questionnaires handed out to the employees, 404 were returned correctly completed (96%). Aged 20 to 52 years, these workers were 340 men and 63 women. The cultural and socioeconomic background was variable, from incomplete primary school to professors with post-graduate degrees, and from manual workers to managing directors. From these 404 workers, 160 persons were identified as presenting headaches (39%), 44 of whom considered it worthwhile answering MIDAS (27.5% of headache sufferers; 10.9% of total workers). Nine of these individuals who answered MIDAS had results of ≥ 21 points and were interviewed on a later date, confirming these results. Three were cases of chronic daily headache (transformed migraine) and six were migraine. The nine cases of chronic and highly disabling migraine are all under treatment under the author's care, free of charge, in keeping with the guarantee personally given to the workers entering this study.

With regard to gender, prevalence of headache was similar in women (31:64) and in men (129:244).

Four individuals with cases of tension type headache who answered MIDAS could be identified by the characteristics of the headache and by an intensity of ≤ 4 points (on a scale from 1 to 10). The other 31 persons with migraine who answered MIDAS rated ≤ 20 points, but in all cases the intensity of the headache was ≥ 5 (1 to 10). For these individuals, it was recommended that medical treatment be sought in the company's medical department. The 116 individuals with headache who did not answer MIDAS considered their headaches to be very occasional and therefore it was not worth answering a specific assessment of headache impact.

## DISCUSSION

MIDAS has been validated, even by retesting, in the USA and UK.^15^ By translating MIDAS into Brazilian Portuguese, we seem to have a useful tool for identifying severe cases of migraine in a working environment where headaches are not usually acceptable excuses for missing work.^16,17^ The workers did not consider MIDAS difficult to answer. Their high level of participation demonstrates that this medical condition was of real interest among the workers, whether they were sufferers or not. Although only 2.2% of all workers entering this study presented severe cases of migraine, identifying and treating these individuals may alter their personal and professional lives.

It is important to observe that MIDAS has usually been validated in specialized headache clinics, with known sufferers of migraine. The possibility of identifying patients in need of treatment, even if they do not know the reason for their constant attacks of headache, may be an extra tool in occupational medicine and general practice.^18-21^ When interviewed individually, a few migraineurs in this study were surprised to understand that the headache they had for years attributed to sinusitis or visual impairment was indeed migraine.

## CONCLUSION

MIDAS in Portuguese may apparently be used by people with a variety of educational and social backgrounds. MIDAS was shown to be a reliable and valuable tool for identifying cases of migraine in some countries. We believe that our Brazilian migraine sufferers may be able to assess their own degree of disability with such simple questionnaire. Guided by the scores in MIDAS, migraine sufferers and their employers should be able to understand the need for treatment.

## NOTES

The full translation of MIDAS into Brazilian Portuguese may be accessed at either of the following sites: planeta.terra.com.br/educacao/neurohomepage/midas.html and www.sbce.med.br/midas.html

The complete translation, as found in the above sites, is given in the table.

**Table:** MIDAS - Migrane Disability Assessment Program

***Questionário de Avaliação da Incapacidade por Enxaqueca***
Este questionário pode ajudar você e seu médico a melhorar o tratamento das suas dores de cabeça.
**Você sofre de dor de cabeça?**
Instruções: por favor responda as seguintes questões sobre TODAS as dores de cabeça que você tenha tido durante **os últimos três meses**. Escreva sua resposta no espaço ao lado de cada questão. Escreva zero se você não teve aquela atividade **durante os últimos três meses.**
*Lembre-se de considerar os últimos 90 dias consecutivos*.

1. Quantos dias de trabalho ou de escola você perdeu nos últimos três meses por causa de suas dores de cabeça?
2. Em quantos dias dos últimos três meses você observou que seu rendimento no trabalho ou na escola estava reduzido pela metade ou mais, devido às suas dores de cabeça? (*Não inclua os dias que você contou na questão 1, onde dia de trabalho ou de aula foi perdido).*
3. Em quantos dias dos últimos três meses você não foi capaz de executar o trabalho de casa por causa de suas dores de cabeça?
4. Em quantos dias dos últimos três meses seu rendimento no trabalho de casa foi reduzido pela metade ou mais devido as suas dores de cabeça? (*Não inclua os dias que você contou na questão 3, onde você não pôde fazer o trabalho de casa).*
5. Em quantos dias dos últimos três meses você perdeu atividades familiares, sociais ou de lazer por causa das suas dores de cabeça? Em quantos dias dos últimos três meses você teve dor de cabeça? *(Se a dor durou mais que um dia, conte cada um dos dias)*. Em uma escala de 0 - 10, em média qual a intensidade da dor destas dores de cabeça? *(0 = nenhuma dor; 10 = dor máxima possível).*
Tendo preenchido este questionário, some os números de dias das questões 1 - 5 (não considere as questões A e B). Se o resultado total for maior que 6, sugerimos que você marque uma consulta com seu médico (leve este questionário com você).
